# Development and validation of a prediction model for long-term cognitive frailty risk in stroke patients based on CHARLS data

**DOI:** 10.1371/journal.pone.0340715

**Published:** 2026-03-25

**Authors:** Shunli Zuo, Ning Liu, Jiaxian Wang, Jingling Li, Xiuyuan Zhu, Yuyang Jia

**Affiliations:** 1 Department of Nursing, Zunyi Medical University Zhuhai Campus, Zhuhai, China; 2 Department of General Practice, Zunyi Medical University Zhuhai Campus, Zhuhai, China; University Hospital of Padova, ITALY

## Abstract

**Background:**

This study aimed to develop and validate machine learning (ML) models for predicting the risk of cognitive frailty in community-dwelling elderly adults with stroke.

**Methods:**

This study involved 2,325 stroke survivors from the China Health and Retirement Longitudinal Study (CHARLS), conducted between 2018 and 2020. We examined 22 behavioral variables, encompassing indicators from the sociodemographic, physical, psychological, cognitive, and social domains. LASSO regression was employed to identify predictive factors, and eight machine learning models—Logistic Regression, Decision Tree, XGBoost, Support Vector Machine, k-Nearest Neighbors, Naïve Bayes, Random Forest, and LightGBM—were utilized to ascertain the optimal model for predicting cognitive frailty among stroke survivors. SHapley Additive exPlanations (SHAP) values were applied to interpret the contributions of the variables.

**Results:**

A total of 2,325 stroke patients were included in the study, among whom 688 (29.59%) exhibited symptoms of cognitive frailty. Of the eight models evaluated, XGBoost (AUC = 0.810) and Random Forest (AUC = 0.795) demonstrated the highest predictive performance for stroke-related cognitive frailty. Key predictors identified were education, nutritional status, physical exercise, Instrumental Activities of Daily Living (IADL), and age, with corresponding SHAP values of 0.28, 0.18, 0.16, 0.21, and 0.32, respectively. The SHAP values indicated that age and education level are the most significant factors in predicting the risk of cognitive frailty in this population.

**Conclusion:**

This study developed eight risk prediction models for post-stroke cognitive frailty utilizing machine learning, with the XGBoost algorithm demonstrating superior performance. Leveraging readily available clinical and demographic indicators, the optimized XGBoost model serves as a practical tool for the early screening of cognitive frailty risk among community-dwelling elderly stroke survivors, particularly within primary care settings. This model can aid clinicians in devising targeted intervention strategies to mitigate disease progression and establish a foundation for future prospective studies examining the mechanisms underlying cognitive frailty in stroke populations. Further external validation is necessary to confirm its generalizability across various clinical contexts.

## Introduction

Cognitive frailty is a heterogeneous syndrome defined by concurrent cognitive impairment and physical frailty in the absence of Alzheimer’s disease or other dementias [1,2]. It is considered potentially reversible and comprises two subtypes: reversible cognitive frailty and potentially reversible cognitive frailty [3]. The interaction between cognitive decline and physical deterioration poses a substantial risk, especially for middle-aged and older adults after stroke [4,5]. Reported prevalence of cognitive frailty after stroke ranges from 50% to 70% [6–8]. During post-stroke rehabilitation, cognitive domains such as attention, memory, and executive function frequently worsen [2,9]. Cognitive frailty impairs independent living and increases risks of depression, anxiety, dependence, reduced quality of life, and higher healthcare costs, thereby imposing additional societal burden [10–14]. Emerging evidence indicates that the reversibility of cognitive frailty depends on its stage, with earlier interventions offering greater potential for recovery [15–18]. Nevertheless, post-stroke rehabilitation remains largely focused on physical recovery, while cognitive assessment and intervention are insufficiently addressed [19]. Consequently, there is an urgent need to clarify risk factors and mechanisms of post-stroke cognitive frailty, develop accurate predictive methods, and design personalized strategies for diagnosis, treatment, and follow-up.

Machine learning (ML) has shown substantial promise in medicine and healthcare because of its ability to recognize patterns and generate predictions [20]. It applies to diverse data types and excels at handling large, high-dimensional, multi-source heterogeneous datasets [21,22]. By extracting latent patterns from complex data, ML models can help optimize individualized treatment strategies and support dynamic monitoring and management of health states. Recently, ML approaches have gained prominence in forecasting complex geriatric syndromes, particularly cognitive disorders. Several models have been developed to identify people at risk of cognitive impairment and dementia. For example, Li et al. [23] developed and validated a risk prediction model for cognitive impairment in older adults; Dong et al. [24] constructed a clinical model for cognitive impairment six months after stroke; and Wei et al. [25] built a machine-learning–based prediction model for poststroke dementia. These efforts have largely targeted cognitive impairment and dementia following stroke. However, few models have been developed to detect the risk of cognitive frailty among stroke survivors in community settings.

This study used data from the China Health and Retirement Longitudinal Study (CHARLS). In the county and village sampling stages, a multi-stage probability-proportional-to-size (PPS) method was applied. The survey encompassed 150 counties across 28 provinces, including autonomous regions and municipalities directly under the central government. A baseline survey was conducted in 2011, with national follow-ups in 2013, 2015, 2018, and 2020. Multidimensional data—psychosocial assessments (CES-D Depression Scale), biomarkers (e.g., cystatin C), and socioeconomic factors—were collected and integrated to enable machine learning (ML) analyses [26]. To date, ML studies using CHARLS have examined cognitive impairment in community-dwelling older adults [27,28]; however, none have developed an interpretable model specifically for predicting post-stroke cognitive frailty. This study aims to fill that critical gap.

Although machine learning (ML) models demonstrate robust predictive performance, the interpretation of individual variable contributions poses significant challenges, hindering their clinical application [29]. The SHapley Additive exPlanations (SHAP) method employs principles of optimal credit allocation and local interpretation, thereby enhancing model interpretability through the visual representation of feature importance [30].Consequently, this study aims to utilize CHARLS data to develop and validate an interpretable ML model and to apply SHAP for visual interpretation, with the objective of accurately and promptly predicting long-term cognitive frailty risk (years post-stroke) in community-dwelling stroke survivors.

## Methods and materials

### Research population

This study used data from the 2018 and 2020 waves of the CHARLS. This study used data from the 2018 and 2020 waves of the China Health and Retirement Longitudinal Study (CHARLS). This study is a retrospective analysis based on data from the CHARLS. The original CHARLS protocol was approved by the Ethics Review Board of Peking University (approval number: IRB00001052–11015), and all participants provided written informed consent at the time of enrollment.

**Fig 1:**
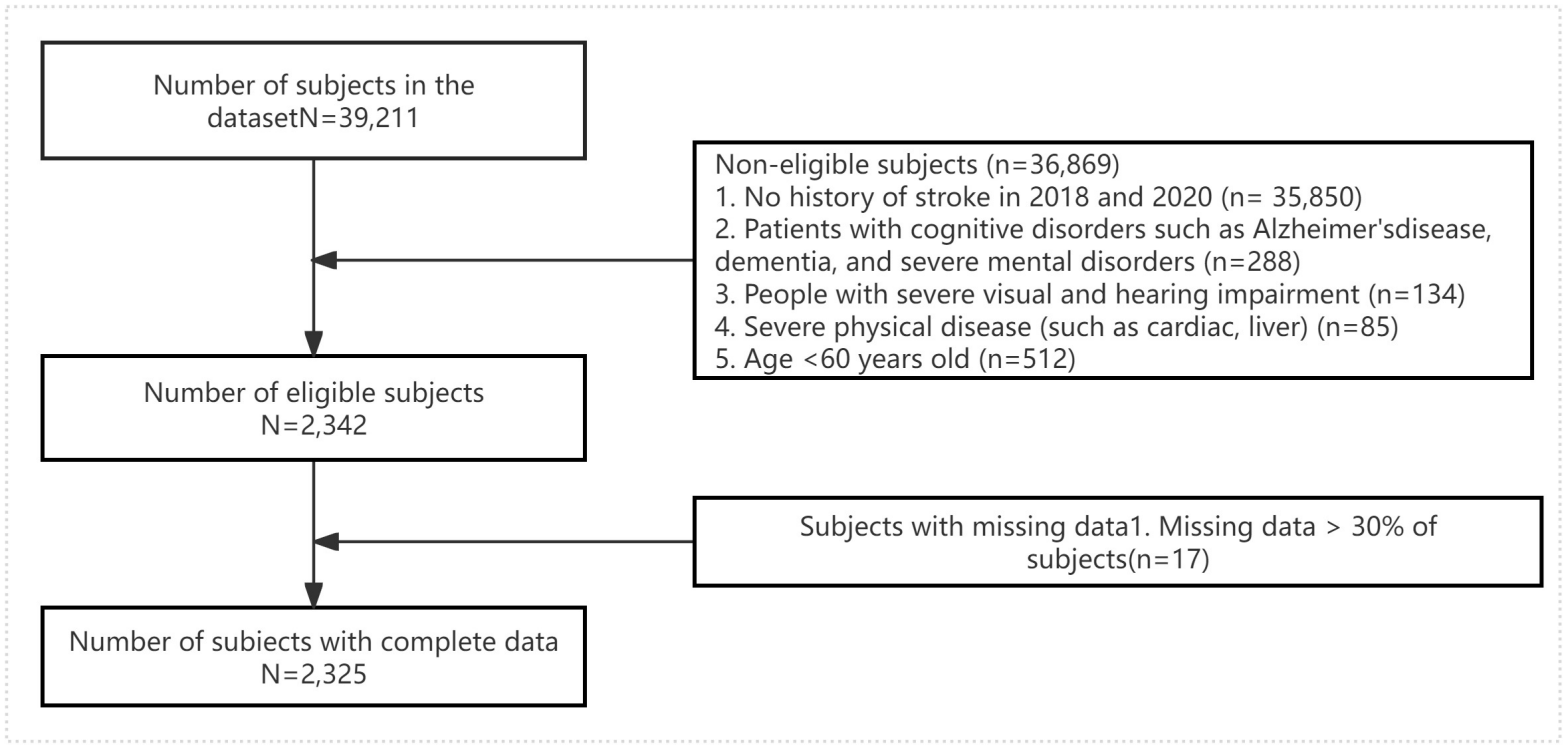
Subject selection flow chart of this study.

We included individuals aged 60 years and older who reported a physician diagnosis of stroke during these survey years. The exclusion criteria were as follows: (1) no history of stroke in 2018 or 2020; (2) diagnosis of Alzheimer’s disease, dementia, or severe psychiatric disorders; (3) severe aphasia or dysarthria that hindered reliable cognitive assessment; (4) significant visual or auditory impairments that interfered with cognitive testing; and (5) severe systemic diseases, such as advanced heart failure or end-stage liver disease. After applying these criteria, a total of 2,325 stroke survivors were included in the final analysis. A flowchart illustrating participant selection, from the original CHARLS sample to the final cohort, is presented in Fig 1.

### Outcome: Cognitive frailty

Cognitive frailty is defined by international consensus as the coexistence of physical frailty and mild cognitive impairment without dementia [1]. Physical frailty was assessed using the Fried frailty phenotype (FP) [31]:consisting of unintentional weight loss, slow walking speed, weak grip strength, self-reported exhaustion, low physical activity. Each component received score 0 (absent) or 1 (present), with scores 0–5. Scores more than 3 were frailty, 1–2 were pre-frailty and score 0 was no frailty. Cognitive impairment was assessed using the Mini-Mental State Examination (MMSE) [32] and the Clinical Dementia Rating (CDR) [33]. Participants were cognitively impaired if they had CDR score less than 18 (no formal education), less than 21 (1–6 years education) or less than 25 (more than 6 years education) were cognitively impaired if they had education-adjusted MMSE score less than 18 (no formal education), less than 21 (1–6 years education) or less than 25 (more than 6 years education). Participants who had Alzheimer’s disease or other dementias (CDR ≥ 1.0) were excluded, as such impairment is severe irreversible cognitive impairment distinct from mild non-demented cognitive impairment required for diagnosis of cognitive frailty.

### Data extraction

During the development phase of the cognitive frailty risk prediction model for stroke survivors, variable screening was informed by a review of the literature [34,35], clinical experience, and PSCI scales [36,37]. Correlation analyses were performed to assess the relationship between variables and cognitive decline. Ultimately, 22 predictive factors were identified from the CHARLS dataset to facilitate a comprehensive evaluation. The same set of variables was employed in the model validation phase to ensure methodological consistency and evaluate predictive stability.

- **Demographic information**: sex, age, education level, residence (urban/rural), living arrangement (alone vs. with others), sleep quality (good vs. poor).
- **Lifestyle and activity indicators**: physical exercise (structured moderate-intensity activities such as square dancing, brisk walking, tai chi, swimming, or ball games ≥2 times/week, ≥ 30 minutes/session, excluding household chores); intellectual activities (e.g., reading, writing, calligraphy, photography, painting, musical instruments, handicrafts, stock trading, card/mahjong games, chess, internet use, or attending university for older adults ≥2 times/week); social activities (visiting or socializing with friends, volunteering, or club participation >2 times/week); smoking; drinking.
- **Health status and self-reported indicators**: chronic pain, history of falls, self-rated life satisfaction, self-rated health, and optimism about the future.
- **Physical and clinical measures** include number of chronic diseases, body mass index (BMI), waist circumference and nutritional status. Nutritional status was assessed using Mini Nutritional Assessment-Short Form (MNA-SF) consisting of food frequency, appetite and weight changes from CHARLS.MNA-SF score below 12 indicates malnutrition, while score above 12 indicates adequate nutrition. Depression was evaluated using 10-item Center for Epidemiologic Studies Depression Scale (CES-D-10) also based on CHARLS. Scores below 10 on CES-D-10 are depressive, while scores below 10 are non-depressive. Instrumental Activities of Daily Living (IADL) capacity was assessed using simplified IADL scale for living scenarios of elderly population. Respondents who reported inability to complete or needed help with at least one of the six IADL items were classified as IADL disability while those who could independently complete all six items were considered intact IADL capacity.

### Data processing

Missing values (≤30%) were imputed using the mice package in R, employing predictive mean matching for continuous variables and logistic regression for categorical variables. Outliers were assessed and adjusted based on clinically acceptable ranges. Continuous variables underwent normalization, while categorical variables were subjected to one-hot encoding. For feature selection, we applied least absolute shrinkage and selection operator (LASSO) regression to the training set. We used 10-fold cross-validation to determine the optimal regularization parameter (λ), following the “1-standard error (1-SE) criterion.” This widely used approach favors model parsimony and predictive stability by choosing the largest λ within one standard error of the minimum cross-validation error [38,39]. To evaluate the robustness of the selected variables, we conducted a sensitivity analysis with elastic net regression using α values from 0.1 to 1, where α = 1 corresponds to LASSO. The analysis confirmed stable predictive contributions of the retained features across regularization schemes. This combined strategy reduces collinearity and overfitting while preserving the most prognostically relevant variables for subsequent model development [40].

### Model development

**Fig 2:**
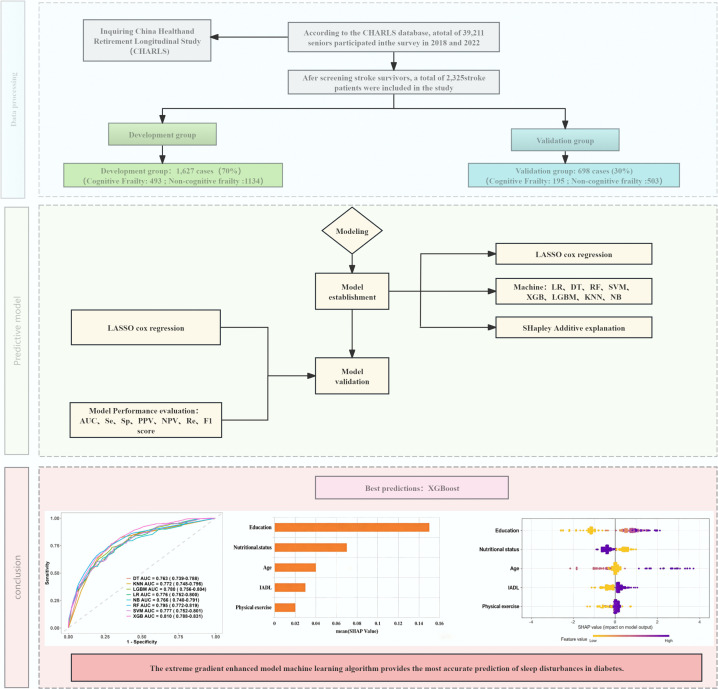
Model construction flowchart. LR: Logistic Regression; DT: Decision Tree;SVM: Support Vector Machine; LGBM:Light Gradient Boosting Machine; XGBoost: EXtreme Gradient Boosting; RF:Random Forest; NB:Naive Bayes; KNN:k-nearest neighbors; AUC:Area Under Curye: SHAP. SHapley Additive explanation; Se:Sensitivity; SP:Specificity; PPV: Pos Pred Value; NPV:Neg Pred Value; Re:Recall.

Eight machine learning algorithms were employed to develop prediction models: logistic regression (LR), decision tree (DT), support vector machine (SVM), extreme gradient boosting (XGBoost), k-nearest neighbors (KNN), naïve Bayes (NB), random forest (RF), and light gradient boosting machine (LightGBM). Participants were randomly assigned to training (70%) and testing (30%) sets through stratified sampling to reduce bias. The models were constructed using the training set and subsequently validated internally with the testing set. All analyses were conducted in R (version 4.4.2). The workflow for model construction is illustrated in **Fig 2**.

We assessed the performance of each model using the test set by generating receiver operating characteristic (ROC) curves and evaluating the predictive model through metrics such as area under the curve (AUC), sensitivity, specificity, recall, F1 score, and accuracy. The F1 score, which evaluates binary models by balancing precision and recall, yields values closer to 1 that indicate superior performance [41]. To improve interpretability, we calculated SHAP values using the “shapforxgboost” R package. These SHAP values quantify the marginal contribution of each predictor to the model’s output by averaging across all possible combinations of predictors. We created SHAP summary plots to visualize overall feature importance and SHAP dependence plots to demonstrate the effects of features on predictions [30].

### Statistical analysis

Normally distributed variables were reported as mean ± standard deviation and analyzed using independent-samples t-tests. Non-normally distributed variables were presented as median (interquartile range) and assessed with the Mann–Whitney U test. Categorical variables were expressed as counts and percentages, with group differences evaluated using chi-square or Fisher’s exact tests. Correlations were analyzed using Pearson correlation coefficients. LASSO logistic regression was performed with the glmnet package, and ROC curves were generated using the pROC package. All statistical analyses were conducted in R software (version 4.4.2), which was selected for its compatibility with the latest stable versions of ML and SHAP-related packages. A two-sided p-value <0.05 was deemed statistically significant.

## Results

### Demographic information about the participants

This study involved 2,325 stroke survivors, among whom 688 (29.59%) were classified as having cognitive frailty (CF). The baseline characteristics of the study population, categorized by CF status, are detailed in Table 1. Participants were randomly assigned to a development set (n = 1,627) and a validation set (n = 698). Within the development set, 493 participants (30.30%) exhibited CF, while the validation set included 195 participants (27.94%) with CF. A comparative analysis of patient characteristics between the two sets is presented in Table 2. In the development set, CF was significantly associated with age, education, sex, marital status, permanent residence, instrumental activities of daily living (IADL), body mass index (BMI), nutritional status, chronic pain, social activity, living arrangement, and physical exercise (all p < 0.05). Similar associations were noted in the validation set.

**Table 1:** Baseline characteristics of the study population.

Characteristic	Total(n = 2,325)	NCF(n = 1,637)	CF (n = 688)	*p value*
Age:				<0.001
60~	1,625 (69.89%)	1151 (70.31%)	474 (68.90%)	
70~	601 (25.85%)	464 (28.34%)	137 (19.91%)	
≥ 80	99 (4.26%)	22 (1.34%)	77 (11.19%)	
Education:				<0.001
below primary school	1,057 (45.46%)	940 (57.42%)	117 (17.01%)	
primary school	559 (24.04%)	360 (21.99%)	199 (28.92%)	
junior high school	437 (18.80%)	220 (13.44%)	217 (31.54%)	
high school and above	272 (11.70%)	117 (7.15%)	155 (22.53%)	
Gender:				<0.001
female	1,138 (48.95%)	860 (52.54%)	278 (40.41%)	
male	1,187 (51.05%)	777 (47.46%)	410 (59.59%)	
Marital.status:				<0.001
unmarried/divorced/ widowed	521 (22.41%)	409 (24.98%)	112 (16.28%)	
be married	1,804 (77.59%)	1,228 (75.02%)	576 (83.72%)	
Permanent.address				<0.001
urban	973 (41.85%)	606 (37.02%)	367 (53.34%)	
rural	1,352 (58.15%)	1,031 (62.98%)	321 (46.66%)	
Self.reported.health. status				0.491
less satisfied/average	77 (3.31%)	51 (3.12%)	26 (3.78%)	
satisfied	2,248 (96.69%)	1,586 (96.88%)	662 (96.22%)	
IADL:				<0.001
normal	1,092 (46.97%)	882 (53.88%)	210 (30.52%)	
impaired	1,233 (53.03%)	755 (46.12%)	478 (69.48%)	
Number.of.chronic. diseases				0.335
0~	431 (18.54%)	301 (18.39%)	130 (18.90%)	
2~	911 (39.18%)	657 (40.13%)	254 (36.92%)	
≥ 4	983 (42.28%)	679 (41.48%)	304 (44.19%)	
Drinking				0.079
no	1,774 (76.30%)	1,266 (77.34%)	508 (73.84%)	
yes	551 (23.70%)	371 (22.66%)	180 (26.16%)	
Smoking				0.090
yes	368 (15.83%)	245 (14.97%)	123 (17.88%)	
no	1,957 (84.17%)	1,392 (85.03%)	565 (82.12%)	
BMI				<0.001
≤ 24	1840 (79.14%)	1358 (82.96%)	482 (70.06%)	
≥ 24	485 (20.86%)	279 (17.04%)	206 (29.94%)	
Nutritional.status				<0.001
malnutrition	945 (40.65%)	525 (32.07%)	420 (61.05%)	
well-nourished	1,380 (59.35%)	1,112 (67.93%)	268 (38.95%)	
Life.satisfaction				0.572
less satisfied/average	98 (4.22%)	66 (4.03%)	32 (4.65%)	
satisfied	2,227 (95.78%)	1,571 (95.97%)	656 (95.35%)	
Chronic.pain				<0.001
no	1,565 (67.31%)	1,056 (64.51%)	509 (73.98%)	
yes	760 (32.69%)	581 (35.49%)	179 (26.02%)	
Sleep.quality				0.009
good	2,064 (88.77%)	1,472 (89.92%)	592 (86.05%)	
poor	261 (11.23%)	165 (10.08%)	96 (13.95%)	
Social.event:				<0.001
yes	2,221 (95.53%)	1,582 (96.64%)	639 (92.88%)	
no	104 (4.47%)	55 (3.36%)	49 (7.12%)	
Intellectual.activity				0.001
yes	2,106 (90.58%)	1,460 (89.19%)	646 (93.90%)	
no	219 (9.42%)	177 (10.81%)	42 (6.10%)	
Live.alone				<0.001
no	408 (17.55%)	249 (15.21%)	159 (23.11%)	
yes	1,917 (82.45%)	1,388 (84.79%)	529 (76.89%)	
Self.rated.life. satisfaction				0.105
less satisfied/average	1,858 (79.91%)	1,323 (80.82%)	535 (77.76%)	
satisfied	467 (20.09%)	314 (19.18%)	153 (22.24%)	
History.of.Falls				0.033
no	1,947 (83.74%)	1,353 (82.65%)	594 (86.34%)	
yes	378 (16.26%)	284 (17.35%)	94 (13.66%)	
Physical exercise				<0.001
no	465 (20.00%)	400 (24.43%)	65 (9.45%)	
yes	1,860 (80.00%)	1,237 (75.57%)	623 (90.55%)	
Depression				0.001
no	2,106 (90.58%)	1,460 (89.19%)	646 (93.90%)	
yes	219 (9.42%)	177 (10.81%)	42 (6.10%)	

CF: Cognitive Frailty; NCF: Non-cognitive frailty.

**Table 2:** Comparison of baseline characteristics between cognitive frailty (CF) and non-cognitive frailty (NCF) groups in training and validation cohorts.

Characteristic	Training set (n = 1,627)			Validation set (n = 698)		
	NCF (n = 1,134)	CF (n = 493)	*p value*	NCF (n = 503)	CF (n = 195)	*p value*
Age:			<0.001			<0.001
60~	787 (69.40%)	336 (68.15%)		364 (72.37%)	138 (70.77%)	
70~	331 (29.19%)	104 (21.10%)		133 (26.44%)	33 (16.92%)	
≥ 80	16 (1.41%)	53 (10.75%)		6 (1.19%)	24 (12.31%)	
Education:			<0.001			<0.001
below primary school	641 (56.53%)	89 (18.05%)		299 (59.44%)	28 (14.36%)	
primary school	267 (23.54%)	144 (29.21%)		93 (18.49%)	55 (28.21%)	
junior high school	145 (12.79%)	155 (31.44%)		75 (14.91%)	62 (31.79%)	
high school and above	81 (7.14%)	105 (21.30%)		36 (7.16%)	50 (25.64%)	
Gender:			<0.001			0.001
female	586 (51.68%)	199 (40.37%)		274 (54.47%)	79 (40.51%)	
male	548 (48.32%)	294 (59.63%)		229 (45.53%)	116 (59.49%)	
Marital.status:			<0.001			0.102
unmarried/divorced/ widowed	288 (25.40%)	77 (15.62%)		121 (24.06%)	35 (17.95%)	
be married	846 (74.60%)	416 (84.38%)		382 (75.94%)	160 (82.05%)	
Permanent.address			<0.001			<0.001
urban	418 (36.86%)	257 (52.13%)		188 (37.38%)	110 (56.41%)	
rural	716 (63.14%)	236 (47.87%)		315 (62.62%)	85 (43.59%)	
Self.reported.health. status			0.839			0.083
less satisfied/average	36 (3.17%)	14 (2.84%)		15 (2.98%)	12 (6.15%)	
satisfied	1098 (96.83%)	479 (97.16%)		488 (97.02%)	183 (93.85%)	
IADL:			<0.001			<0.001
normal	615 (54.23%)	153 (31.03%)		267 (53.08%)	57 (29.23%)	
impaired	519 (45.77%)	340 (68.97%)		236 (46.92%)	138 (70.77%)	
Number.of.chronic. diseases			0.891			0.080
0~	216 (19.05%)	91 (18.46%)		85 (16.90%)	39 (20.00%)	
2~	440 (38.80%)	188 (38.13%)		217 (43.14%)	66 (33.85%)	
≥ 4	478 (42.15%)	214 (43.41%)		201 (39.96%)	90 (46.15%)	
Drinking			0.578			0.022
no	865 (76.28%)	369 (74.85%)		401 (79.72%)	139 (71.28%)	
yes	269 (23.72%)	124 (25.15%)		102 (20.28%)	56 (28.72%)	
Smoking			0.246			0.242
yes	179 (15.78%)	90 (18.26%)		66 (13.12%)	33 (16.92%)	
no	955 (84.22%)	403 (81.74%)		437 (86.88%)	162 (83.08%)	
BMI			<0.001			<0.001
≤ 24	942 (83.07%)	352 (71.40%)		416 (82.70%)	130 (66.67%)	
≥ 24	192 (16.93%)	141 (28.60%)		87 (17.30%)	65 (33.33%)	
Nutritional.status			<0.001			<0.001
malnutrition	378 (33.33%)	298 (60.45%)		147 (29.22%)	122 (62.56%)	
well-nourished	756 (66.67%)	195 (39.55%)		356 (70.78%)	73 (37.44%)	
Life.satisfaction			0.440			1.000
less satisfied/average	42 (3.70%)	23 (4.67%)		24 (4.77%)	9 (4.62%)	
satisfied	1,092 (96.30%)	470 (95.33%)		479 (95.23%)	186 (95.38%)	
Chronic.pain			<0.001			0.012
no	731 (64.46%)	363 (73.63%)		325 (64.61%)	146 (74.87%)	
yes	403 (35.54%)	130 (26.37%)		178 (35.39%)	49 (25.13%)	
Sleep.quality			0.006			0.622
good	1,025 (90.39%)	422 (85.60%)		447 (88.87%)	170 (87.18%)	
poor	109 (9.61%)	71 (14.40%)		56 (11.13%)	25 (12.82%)	
Social.event:			0.001			0.114
yes	1,095 (96.56%)	456 (92.49%)		487 (96.82%)	183 (93.85%)	
no	39 (3.44%)	37 (7.51%)		16 (3.18%)	12 (6.15%)	
Intellectual.activity			0.002			0.156
yes	1,016 (89.59%)	466 (94.52%)		444 (88.27%)	180 (92.31%)	
no	118 (10.41%)	27 (5.48%)		59 (11.73%)	15 (7.69%)	
Live.alone			<0.001			0.005
no	170 (14.99%)	110 (22.31%)		79 (15.71%)	49 (25.13%)	
yes	964 (85.01%)	383 (77.69%)		424 (84.29%)	146 (74.87%)	
Self.rated.life. satisfaction			0.068			0.877
less satisfied/average	929 (81.92%)	384 (77.89%)		394 (78.33%)	151 (77.44%)	
satisfied	205 (18.08%)	109 (22.11%)		109 (21.67%)	44 (22.56%)	
History.of.Falls			0.146			0.106
no	936 (82.54%)	422 (85.60%)		417 (82.90%)	172 (88.21%)	
yes	198 (17.46%)	71 (14.40%)		86 (17.10%)	23 (11.79%)	
Physical exercise			<0.001			<0.001
no	273 (24.07%)	42 (8.52%)		127 (25.25%)	23 (11.79%)	
yes	861 (75.93%)	451 (91.48%)		376 (74.75%)	172 (88.21%)	
Depression			0.002			0.156
no	1,016 (89.59%)	466 (94.52%)		444 (88.27%)	180 (92.31%)	
yes	118 (10.41%)	27 (5.48%)		59 (11.73%)	15 (7.69%)	

CF: Cognitive Frailty; NCF: Non-cognitive frailty.

### Model development and performance

**Fig 3:**
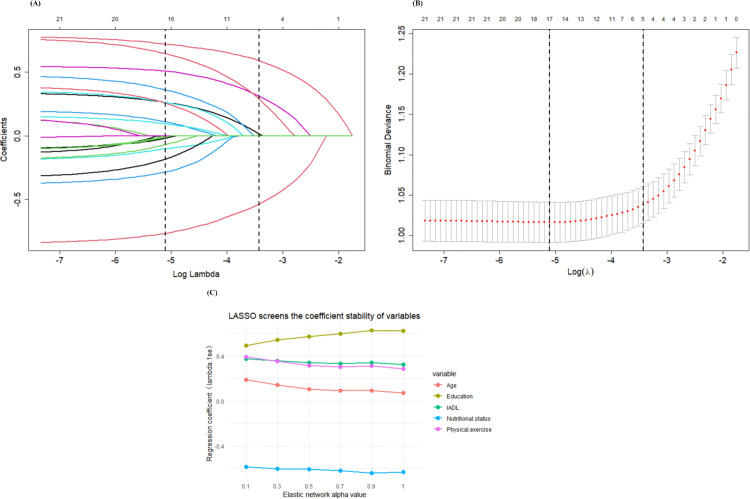
Results of the LASSO regression model for the selection of demographic and clinical characteristics. **(A)** Coefficient curve vs. lambda: Shows coefficient changes with lambda; non-zero coefficients (optimal lambda) indicate key CF prediction features. **(B)** Optimal lambda in LASSO: Log(lambda) vs. partial likelihood deviation curve; optimal lambda (1-SE criterion) identified via tenfold cross-validation (vertical dashed line). **(C)** Coefficient stability across elastic net α: Trends of LASSO-retained variables’ coefficients across α (0.1–1, α = 1 = LASSO) in elastic net regression.

We developed a prediction model utilizing LASSO regression on 22 candidate variables. Employing the 1-standard-error (1-SE) criterion derived from 10-fold cross-validation, we identified five predictors: education, nutritional status, physical exercise, instrumental activities of daily living (IADL), and age. In the LASSO coefficient profile (Fig 3 A) and the cross-validation curve (Fig 3 B), these variables exhibited nonzero coefficients across a spectrum of regularization strengths, demonstrating their sustained contributions to prediction and affirming the robustness of their selection. To assess stability, we conducted a sensitivity analysis using elastic net regression across α values ranging from 0.1 to 1, where α = 1 corresponds to LASSO. The coefficient stability plot (Fig 3C) reveals consistent coefficient trajectories for the selected variables across all α values, without sign reversals or significant magnitude shifts. The overlap rate between LASSO-selected variables and those identified by elastic net exceeded 80% across α values, suggesting that the reduction from 22 candidates to five reflects consistent predictive relevance rather than random fluctuation.

**Fig 4:**
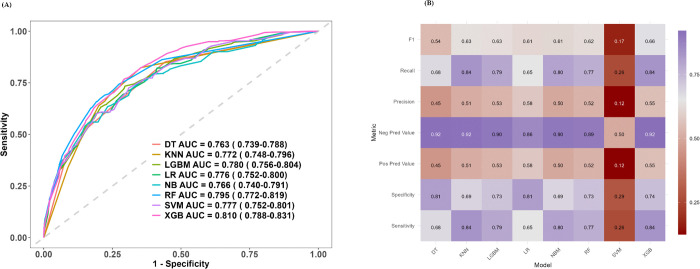
ROC curves and performance of eight machine learning models for predicting CF in the testing set. **(A)** ROC curve. **(B)** heat map Se:Sensitivity. SP:Specificity; PPV: Pos Pred Value;NPV:Neg Pred Value;Re:Recall;LR: Logistic Regression; DT: Decision Tree;SVM: Support Vector Machine; LGBM:Light Gradient Boosting Machine; XGBoost: EXtreme Gradient Boosting; RF:Random Forest; NB:Naive Bayes; KNN:k-nearest neighbors.

Using these predictors, various machine learning models were developed, including logistic regression (LR), decision tree (DT), XGBoost, support vector machine (SVM), k-nearest neighbors (KNN), naive Bayes (NB), random forest (RF), and LightGBM. The receiver operating characteristic (ROC) curves for all models are presented in Fig 4(A). Among these models, XGBoost achieved the highest predictive performance, with an area under the curve (AUC) of 0.810 (95% CI: 0.788–0.831), followed by RF (AUC = 0.795, 95% CI: 0.772–0.819), LightGBM (AUC = 0.780, 95% CI: 0.756–0.804), SVM (AUC = 0.777, 95% CI: 0.752–0.801), LR (AUC = 0.776, 95% CI: 0.752–0.800), KNN (AUC = 0.772, 95% CI: 0.748–0.796), NB (AUC = 0.766, 95% CI: 0.740–0.791), and DT (AUC = 0.763, 95% CI: 0.739–0.788). Model accuracy, sensitivity, and specificity are illustrated in Fig 4(B)and detailed in Table 3. XGBoost demonstrated the best overall performance, achieving the highest accuracy of 0.810, with a sensitivity of 0.84 and a specificity of 0.74, indicating a robust balance between identifying true cases of CF and minimizing false positives.

**Table 3:** Performances of various prediction models predicting CF using a testing data set.

	F1	Recall	Precision	NPV	PPV	Specificity	Sensitivity
DT	0.54	0.68	0.45	0.92	0.45	0.81	0.68
KNN	0.63	0.84	0.51	0.92	0.51	0.69	0.84
LightGBM	0.63	0.79	0.53	0.90	0.53	0.73	0.79
LR	0.61	0.65	0.58	0.86	0.58	0.81	0.65
NBM	0.61	0.80	0.50	0.90	0.50	0.69	0.80
RF	0.62	0.77	0.52	0.89	0.52	0.73	0.77
SVM	0.17	0.26	0.12	0.50	0.12	0.29	0.26
XGBoost	0.66	0.84	0.55	0.92	0.55	0.74	0.84

LR: Logistic Regression; DT: Decision Tree;SVM: Support Vector Machine; LightGBM:Light Gradient Boosting Machine; XGBoost: EXtreme Gradient Boosting; RF:Random Forest; NB:Naive Bayes; KNN:k-nearest neighbors; CF: Cognitive Frailty.

### Predictor importance and SHAP analysis

**Fig 5:**
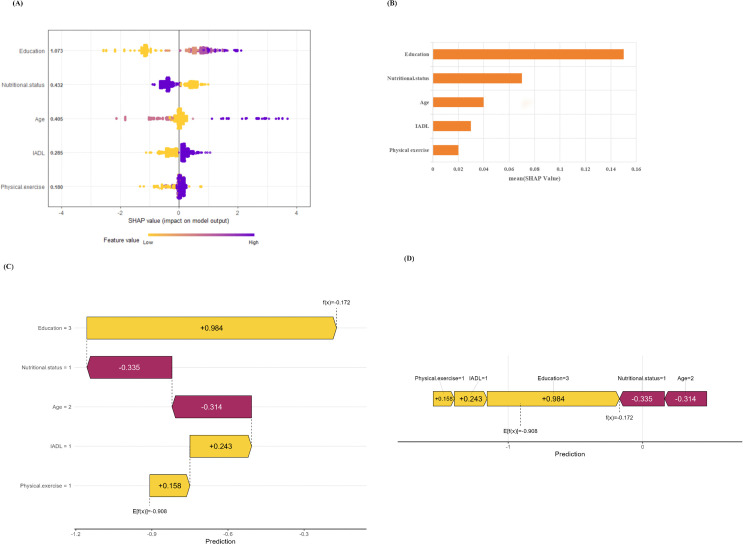
Explanation of the global model using SHAP method. **(A)** XGBoost model feature importance ranking bar plot. **(B)** XGBoost model summary plot. **(C)** XGBoost model waterfall plot. **(D)** The XGBoost model force plot; Each bar represents a feature. Purple indicates a negative contribution to the prediction result (reducing risk), while yellow indicates a positive contribution to the prediction result (increasing risk).

To enhance interpretability, SHAP analysis was conducted on the XGBoost model, and the results are shown in Fig 5. The feature importance ranking plot (Fig 5A) presents predictors in descending order of their impact: education (1.073), nutritional status (0.432), age (0.405), IADL (0.285), and physical exercise (0.180). These findings suggest that education has the most significant influence on predicting cognitive frailty, followed by nutritional status, while physical exercise exerts the least impact among the five predictors. The summary plot (Fig 5B) illustrates the relationship between each predictor and the model output. It shows that higher education and improved nutritional status (indicated in purple, representing negative contributions) correlate with a lower predicted risk of cognitive frailty, whereas advanced age, impaired IADL, and insufficient physical exercise (indicated in yellow, representing positive contributions) elevate the predicted risk. To demonstrate individual-level predictions, a waterfall plot (Fig 5C) and a force plot (Fig 5D) are provided for a representative non-cognitive frailty case. In this instance, the model’s base value (the average prediction across all training samples) was −0.908. Education (value = 3) contributed the largest negative impact (−0.984), significantly reducing the predicted risk, followed by nutritional status (value = 1, contribution = −0.335). Conversely, physical exercise (value = 1), IADL (value = 1), and age (value = 2) had positive contributions (+0.158, + 0.243, + 0.314, respectively), slightly increasing the risk. Ultimately, the cumulative effect of these predictors resulted in a final predicted value of −0.172, indicating a low probability of cognitive frailty development in this individual.

## Discussion

This study developed and validated a machine learning model, specifically the extreme gradient boosting algorithm, to predict the long-term risk of cognitive frailty among middle-aged and older stroke survivors in China, using data from the China Health and Retirement Longitudinal Study. Our model demonstrated robust predictive performance and identified several key predictors, including age, education, nutritional status, physical exercise, and IADL. These findings contribute to the existing literature on the application of machine learning techniques in geriatric risk stratification and the prediction of cognitive frailty.

Currently, the majority of studies on machine learning prediction models related to post-stroke cognitive frailty primarily concentrate on post-stroke cognitive impairment or frailty. In contrast, research specifically addressing cognitive frailty, particularly within community settings for stroke survivors, remains relatively limited.Hu et al. (n = 6718) utilized data from the CLHLS to develop four machine learning models: logistic regression, random forest, XGBoost, and Bayesian networks, aimed at predicting the risk of cognitive impairment among cognitively normal elderly individuals in the Chinese community [42]. Their findings indicated that the combination of Bayesian networks and random forests, utilizing four selected predictors, achieved the highest accuracy, reaching 0.834. In 2023, Ji et al. (n = 397) [43] developed nine machine learning models to predict post-stroke cognitive impairment, identifying the Gaussian Naive Bayes (GNB) model as the most effective, with an AUC of 0.919. Lee et al. [44] also constructed multiple machine learning models to assess the risk of post-stroke cognitive impairment (PSCI) in patients with acute ischemic stroke (AIS). While these models primarily focused on PSCI, it is important to note that cognitive frailty and PSCI, despite potential overlap in clinical practice, have distinct differences in definition, severity, treatment strategies, and intervention methods. As a pre-dementia state, the early identification and accurate prediction of cognitive frailty are crucial for implementing preventive and intervention measures, thereby reducing the risk of cognitive impairment [1]. To our knowledge, studies that specifically develop and interpret machine learning models targeting cognitive vulnerability in post-stroke populations, particularly using large-scale community datasets such as CHARLS, remain relatively scarce.

The SHAP summary graph effectively illustrates the significance of each variable in predicting disability, thereby enhancing the model’s transparency and interpretability. The SHAP chart reveals that education and nutritional status are the two most critical factors. Their SHAP values exhibit a broad distribution range, suggesting that variations in these variables can substantially influence the risk of cognitive frailty. Data from this study indicate that elderly stroke patients face an elevated risk of cognitive frailty when their educational attainment is low. Empirical analysis shows [45] that educational level, a key indicator of cognitive reserve capacity, positively correlates with the preservation of cognitive function post-stroke. Additionally, a meta-analysis indicates [46] that individuals with more than 12 years of education have a 38% lower risk of post-stroke cognitive impairment (PSCI) compared to those with fewer than 6 years of education. Consequently, targeted screening and cognitive interventions are essential for individuals with low educational levels.In the same city, malnutrition may significantly contribute to the pathogenesis of cognitive frailty in elderly stroke patients. A foreign study [47] involving 5,414 community-dwelling individuals aged 55 and older without dementia examined the relationship between nutritional status and cognitive frailty, revealing a notably high prevalence of malnutrition among the elderly exhibiting cognitive frailty. A meta-analysis further indicates [48] that the risk of cognitive frailty in malnourished elderly individuals is 3.06 times greater than that of their well-nourished counterparts. Previous research has also demonstrated [49] that nutrients such as folic acid, flavonoids, and vitamin D exert a protective effect on cognitive function in the elderly, with deficiencies in these nutrients significantly heightening the risk of cognitive impairment. Stroke patients often experience inflammation due to the disease and inadequate nutrient intake, leading to a decline in subcutaneous fat and muscle mass, which markedly increases the risk of frailty [50]. Consequently, it is imperative to closely monitor the nutritional status of stroke patients. The regular application of mini-nutritional assessment (MNA) tools, along with timely dietary support, can play a crucial role in mitigating cognitive frailty.

Regular exercise and the maintenance of functional abilities are inversely related to cognitive frailty. An intervention study involving elderly individuals with cognitive frailty demonstrated that moderate exercise can enhance both cognitive and physical functions in this population [51]. Previous research has indicated that exercise promotes brain remodeling, delays brain atrophy, and improves muscle function, thereby indirectly mitigating cognitive decline [52]. Engaging in scientifically guided exercise holds significant potential for enhancing cognitive function and addressing physical frailty. Additionally, studies reveal that patients with mild cognitive impairment (MCI) frequently encounter challenges in performing complex daily activities, such as financial management and medication adherence, and a decline in these capabilities may signal an increased risk of dementia [53,54]. Consequently, rehabilitation following a stroke should prioritize the recovery of both instrumental activities of daily living and cognitive function, while promoting daily engagement to help postpone cognitive frailty. Age is another critical factor; data indicate that approximately 30% of stroke patients over 65 years old experience cognitive decline within three months of onset, with this figure rising to 50% among those aged 80 and above [55,56]. The interplay of stroke and advanced age may intensify cognitive frailty, leading to a synergistic amplification effect. In clinical practice, it is essential to enhance cognitive monitoring of elderly stroke patients. Standardized tools, such as the Montreal Cognitive Assessment (MoCA), should be integrated into routine evaluations to promote the early identification and intervention of cognitive frailty.

### Clinical and research significance

At the clinical level, this model functions as an early risk-screening tool that assists community and clinical staff in identifying stroke survivors at high risk for cognitive frailty, thereby facilitating timely intervention. For patients with low educational attainment, malnutrition, reduced physical activity, or other functional declines, clinicians can implement targeted multidimensional measures, including nutritional support, cognitive training, and exercise guidance. At the research level, this study establishes a foundation for enhancing the understanding and management of poststroke cognitive frailty. By employing explainable machine learning for long-term risk prediction among stroke survivors in Chinese communities, this work addresses a specific research gap for this population. The model combines robust predictive performance with interpretable outputs, and techniques such as SHAP elucidate the direction and relative importance of key predictors while generating hypotheses for mechanistic studies. Furthermore, the use of a large, nationally representative longitudinal cohort enhances the model’s external applicability and the generalizability of the findings, providing a methodological reference for related research.

### Research limitation and future research directions

Although we use a nationally representative dataset and advanced machine learning techniques, there are some limitations. First, the definition of cognitive frailty is derived from the Fried phenotype and MMSE/CDR scores, which may not comprehensively capture its multifaceted nature, including physical function, neurobiological changes, and psychosocial factors. Second, the inability to differentiate between various stages of cognitive frailty may hinder the precise identification of intervention windows for high-risk groups. Third, the CHARLS database does not include critical variables such as stroke severity, lesion location, and onset time. Additionally, the reliance on self-reported stroke history, which lacks imaging verification, may result in the underreporting of minor strokes, inaccurate reporting of other cerebrovascular events, and recall bias. Fourth, the absence of acute phase data within several months following a stroke limits the model’s applicability, rendering it suitable only for long-term cognitive frailty risk screening, rather than acute phase prediction. Future research should prioritize the collection of acute phase data to develop a staging prediction model. Furthermore, variables such as stroke subtypes and locations were excluded from the analysis due to insufficient sample size.Fifth, this study employed a cross-sectional design. While the association between variables and cognitive frailty was identified, a causal relationship could not be established, and unmeasured confounding factors may exist. Future cohort studies are necessary to further elucidate the causal pathways between predictors and cognitive frailty. Sixth, the model has undergone only internal validation and has yet to be evaluated externally. Consequently, caution is warranted when applying it to populations outside of China or specific groups, such as stroke patients in institutional care; external validation studies should be conducted subsequently. Finally, due to the limitations inherent in cross-sectional data and self-reported measurements, inferring causal relationships remains challenging despite the provision of risk estimates. Future research should aim to verify stroke diagnoses and explore related mechanisms by integrating medical records with imaging data.

## Conclusion

Our research results show that machine learning models, especially XGBoost, can effectively predict cognitive frailty in community environments. The main predictors of cognitive frailty include educational attainment, nutritional status, exercise, IADL and age. However, this model was only developed for the elderly stroke population in the community, and the clinical value of its prediction results in guiding intervention still needs further verification.
